# Development of an Alternative Manufacturing Technology for Niobium Components

**DOI:** 10.3390/ma17133093

**Published:** 2024-06-24

**Authors:** Anna Kawalek, Kirill Ozhmegov, Dariusz Garbiec, Henryk Dyja, Alexandr Arbuz

**Affiliations:** 1 Faculty of Production Engineering and Materials Technology, Częstochowa University of Technology, ul. J.H. Dąbrowskiego 69, 42-201 Częstochowa, Poland; anna.kawalek@pcz.pl (A.K.); kvozhmegov@wp.pl (K.O.); 2 Łukasiewicz Research Network, Poznań Institute of Technology, 6 Ewarysta Estkowskiego St., 61-755 Poznan, Poland; dariusz.garbiec@pit.lukasiewicz.gov.pl (D.G.); henryk.dyja48@gmail.com (H.D.); 3 Research Facility, AEO Nazarbayev University, 53 Kabanbay Batyr Ave, Nur-Sultan 010000, Kazakhstan

**Keywords:** powder metallurgy, Nb rods, SPS methods, sintering parameters, metallography, EBSD, rheological and strength properties

## Abstract

Due to their physical and mechanical properties, niobium products are used in the nuclear power industry, chemical industry, electronics, medicine and in the defence industry. Traditional manufacturing technology for these products is characterized by long production cycles and significant material losses during their surface machining. This paper presents the results of a study on the fabrication of niobium products by Spark Plasma Sintering (SPS). Structural and mechanical tests were conducted on the products obtained, as well as a comparative analysis with the properties of products obtained using traditional technology. Based on the analysis of the test results obtained, recommendations were made for the sintering of Nb powders. It was found that the optimum temperature for sintering the powder is 2000 °C as the density of the material obtained is close to the theoretical density. The microstructure obtained is comparable to samples obtained by the traditional method after recrystallization annealing. Samples obtained according to the new technology are characterized by higher mechanical properties R_p0.2_ and R_m_ and the highest hardness.

## 1. Introduction

Niobium products, as innovative products, have applications in many fields, including those related to nuclear energy, the manufacture of chemical instruments and electronic device components, medicine and the defence industry [1,2,3,4,5,6,7,8,9]. Of the pure metals, niobium has the highest superconductivity temperature (T = 30 K). Niobium-based superconducting materials are used in particle accelerators, including the Large Hadron Collider (LHC) and will be used in the next generation of accelerators (Future Circular Collider, LCC). These materials provide a critical current density of up to 3500 A/mm^2^ in a magnetic field of 12 T and at a temperature of 4.2 K. They are characterized by low residual resistivity and good mechanical properties [10,11]. Niobium is also used in the nuclear power industry due to its small effective neutron absorption cross-section (1.15 × 10^−28^ m^2^) and good tensile strength. These favorable properties determine niobium and niobium matrix alloys as the materials of choice for fuel rod cladding in nuclear reactors [5,6].

Niobium is the main element for doping zirconium in the manufacture of shrouds and fuel rods for pressurized water reactors (e.g., PWR) [11,12,13,14]. In these alloys, niobium increases corrosion resistance and effectively reduces hydrogen content [12]. Niobium and its alloys are increasingly replacing tantalum in the manufacture of jet engine components, with niobium being preferred over tantalum as it has a much lower specific mass and is more amenable to plastic processing [6]. Niobium can be used for implants due to its high biocompatibility [3,4]. Pure niobium and its oxide are also used in the construction of capacitors, replacing tantalum in systems with special requirements. In recent years, niobium has also been used for the catalytic conversion of palm oil, used as a biofuel for diesel engines [2,6].

The traditional technological process for manufacturing niobium products encompasses the following operations [6,15,16,17,18,19]:

− Production of sintered niobium powder by pressing and sintering methods;
− Melting the sinter in arc furnaces to produce an ingot;
− Obtaining a semi-finished product by hot forging of ingots and its machining;
− Hot bar extrusion and machining;
− Manufacture of finished products using cold forming methods (rolling, drawing) in combination with various intermediate operations.

The process for manufacture of niobium products or semi-finished products is very complex, due to the large number of forming processes and intermediate heat treatment and machining operations. The efficiency of these processes is low, as is the yield, due to the high process waste in individual operations.

The method for producing semi-finished products from niobium powder involves pressing niobium powder at 710 MPa, followed by sintering using a direct current. This process is conducted in vacuum conditions (no less than 1 × 10^−5^ Torr). After the sintering process, the metal is subjected to a cold forging process with a relative crumple of up to 20% and then sintered again [6].

There is also a known method whereby rods measuring 0.025 × 0.65 × 1.2 m are pressed from Nb powder and sintered in a vacuum furnace. The vacuum system provides a pressure of at least 1 × 10^−5^ Torr. Up to a temperature of T = 300–400 °C, the heating of the rods takes place at a slow rate. This is followed by a holding process to remove hydrogen, after which the temperature is raised to 2300 °C. The heating time is approximately 40 min. CO removal takes place at 1650 °C, accompanied by a significant pressure increase in the vacuum system. The maximum temperature is maintained for 2–10 h, depending on the rod size [6].

SPS sintering technology is one of the most modern methods for consolidating powdered materials such as metallic powders, ceramic powders, composite powders, carbides, nitrides, borides, fluorides and others [20,21,22,23,24,25,26,27,28,29,30]. In the SPS method, sintering with simultaneous pressing takes place in a closed working chamber in an atmosphere of nitrogen, argon, hydrogen or vacuum. Unlike other sintering methods, in which the powder is heated using alternating current, this method uses periodically repeated pulses of direct current, lasting from a few to several hundred milliseconds, with low voltage but high amperage (from a few to several thousand amperes) to heat the consolidated powder. The SPS process is characterized by high efficiency due to the direct supply of energy to the sintered powder, without its loss to ambient heating. The energy used in the SPS process is much higher than in other methods, so the main advantages of sintering using high-current pulses of direct current are the short duration of the process (several to several minutes) and its operation at much lower temperatures (up to 30% lower than conventional sintering) [31]. The rapid heating and cooling (up to 1000 °C/min) and short sintering time protect the grains from excessive growth so that the structure of the starting material is preserved in the sintered rod.

According to the authors of [32], the SPS sintering process can be divided into the following stages:

(1) Activation and cleaning of the surface of the powder particles;
(2) Formation of “necks” between the particles;
(3) Growth of the resulting “necks”;
(4) Compaction of the material by plastic deformation.

This paper presents a method for obtaining niobium blanks in a single process by Spark Plasma Sintering (SPS) [33]. The obtained semi-finished products will have a homogeneous recrystallized microstructure and high strength properties.

## 2. Research Material and Methods

This study involved the use of niobium powder, the chemical composition of which is shown in Table 1. Powder particle sizes were in the range of 40–63 µm.

The niobium powder was sintered in an HP D 25/3 SPS furnace (FCT Systeme, Rauenstein, Germany). Sintering was performed using tools made of grade 2333 graphite (Mersen, Gennevilliers, France). The powder was separated from the tools with a tantalum film, followed by a graphite film. The tantalum foil was used to protect the niobium powder from reacting with graphite tool carbon, which adversely affects corrosion resistance [6]. The foil was also chosen because of the sintering temperature of the niobium powder. The spark-plasma sintering process was conducted in a vacuum of 5 × 10^−2^ mbar. The pre-tightening force was 5 kN (it is an initial load at the beginning of the process which is applied in HP D 25/3 (FCT Systeme)). In the feed chamber, niobium powder was subjected to the following processes: pressing at 50 MPa, the pressure was maintained throughout the process, followed by heating using pulsed current to a sintering temperature of 1800–2200 °C, with a heating rate of 100 °C/min. The pulse duration was 125 ms and the break time between pulses was 5 ms. Sintering at the sintering temperature for 10 min and cooling to ambient temperature at a cooling rate of 400 °C/min.

Sinters with dimensions of Ø40 × 12 mm, Ø30 × 11 mm and Ø20 × 30 mm were obtained as a result of the sintering process. The density of the obtained sinter was determined using a laboratory scale (Ohaus) equipped with the necessary equipment.

Density measurements were conducted in accordance with the PN-EN ISO 2738:2001 standard [34] according to dependency 1.

(1) $$\rho =\frac{m_{1}\times g_{w}}{m_{3}-m_{4}}$$

where:

*m*_1_—dry sample mass;
*m*_3_—mass of sample weighed above water after soaking;
*m*_4_—mass of sample weighed in water after soaking;
g_w_—water density, dependent on ambient temperature.

Vickers hardness measurements were performed according to ISO 6507-1:2007 [35] using a Future Tech FM-700 (Cedar Park, TX, USA) microhardness gauge at a load of 0.05 HV.

A metallographic study was carried out on a CrossBeam-540 scanning electron microscope (Carl Zeiss, Oberkochen, Germany). The phase composition was evaluated by backscattered electron diffraction (EBSD) using a NordlysNano detector (Oxford Instruments, Abingdon, UK) and AZtec, HKL Channel5 and Tango software v.5.12.74.0 (Oxford Instruments, Abingdon, UK).

Sample preparation for the SEM/EBSD study cycle included cutting in half on a Brilliant 220 (San Francisco, CA, USA) precision cutting machine (QATM) in diameter at a low speed of 20 μm/s and intensive water cooling, then the workpiece was ground and polished on an automatic Saphir 520 (QATM) machine (QATM, Mammelzen, Germany). The final polishing was carried out electrolytically (jet polishing) using a LectroPol-5 (Struers) machine (Struers, Rotherham, UK). This operation is especially important for the accurate removal of all residues of the layer amorphized by mechanical grinding and polishing, making it possible to obtain diffraction patterns of the Kikuchibands for EBSD.

To determine the rheological and strength properties of the resulting material, tests were carried out using a Gleeble 3800 plastomer (Poestenkill, NY, USA), involving swell tests on cylindrical samples. The strain parameters of the test samples are shown in Table 2, while the methodology for conducting these tests is described in works [36,37,38,39,40,41,42,43].

The sintered samples were subjected to thermal treatment in vacuum at T = 1100 °C for 30 min to determine the effect of recrystallization annealing on material properties. This annealing method ensures the formation of a recrystallized sample structure [6].

The test result analysis was conducted with reference to previously published results of niobium obtained according to traditional technology [6,15,16,17,18,19].

## 3. Results and Discussion

### 3.1. Study of the Impact of Nb Powder Sintering Parameters on the Density and Hardness of the Resulting Sample

**Figure 1:**
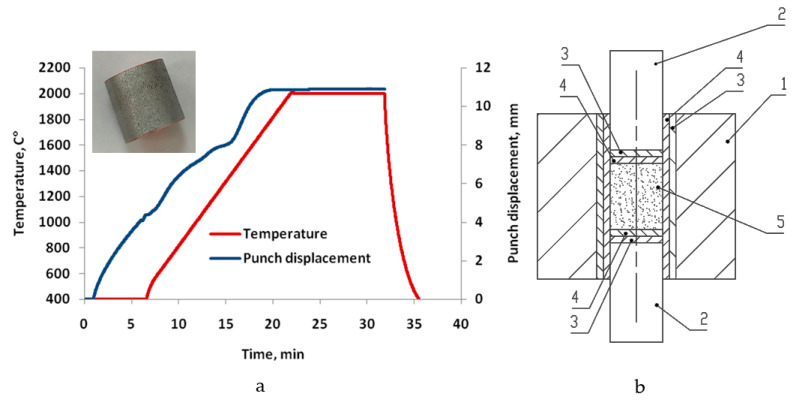
Sintering curves of niobium powder spark plasma sintered at 2000 °C niobium sample with dimensions Ø20 × 30 mm (**a**) and schema (**b**) of the tool system for producing semi-finished products from niobium powder by spark plasma sintering (SPS).

Figure 1 shows an example of the dependence of temperature and sample height variation in the sintering of Nb powder using the SPS method.

The sample was heated at a constant rate of 100 °C/min to a sintering temperature of 2000 °C; the sample sintering time was 10 min. After sintering, the sample was cooled at a rate of 400 °C/min. A constant pressure of 50 MPa was applied during pressing. The data shown in Figure 1a show that upon reaching a sintering temperature of 2000 °C, an adequate density of the sintered powder is achieved, as a ‘plateau’ effect can be observed in the curves illustrating the densification process. The resulting material density is almost equal to the theoretical density (ρ = 8.57 g/cm^3^). A slight movement of the punch at the end of the sintering process means that residual porosity is eliminated.

Figure 1b shows a schema of the tool system for producing semi-finished products from niobium powder by spark plasma sintering (SPS), in axial section (1—die; 2—upper and lower punches; 3—graphite foil; 4—tantalum foil; 5—powder niobium).

The results of density and hardness tests on Nb samples obtained at different sintering temperatures are shown in Table 3.

As the samples sintered at T < 1800 °C were of significantly lower density and hardness, they were not included in further studies.

Data in Table 3 lead to the conclusion that the optimum sintering temperature for Nb powder is 2000 °C, as the samples obtained at this temperature have the highest hardness and a density almost equal to the theoretical density.

Although similar results were obtained after sintering Nb powder at 2200 °C, sintering at this temperature leads to a manufacturing cost increase due to a rise in energy consumption.

### 3.2. Study of the Impact of Nb Powder Sintering Parameters on the Resulting Sample Structure

**Figure 2:**
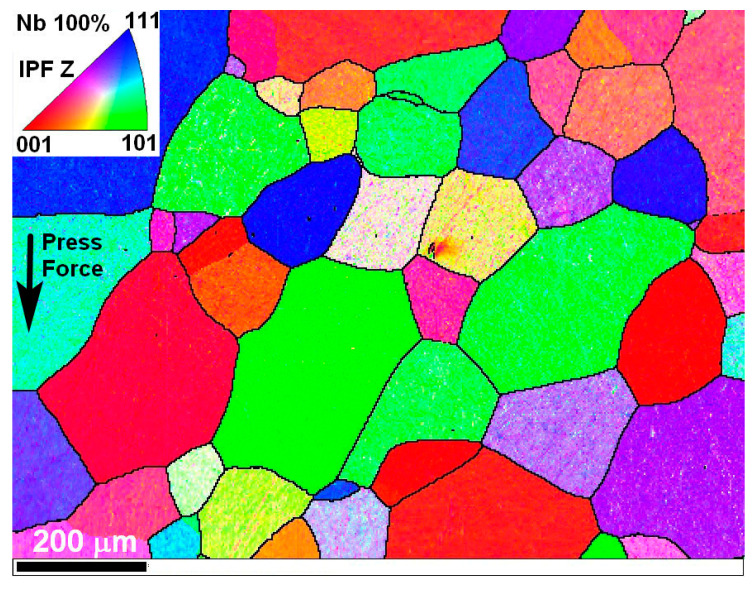
Structural test results of the Nb sample after SPS sintering (sintering temperature T = 2000 °C) obtained with CrossBeam-540 (Carl Zeiss, Oberkochen, Germany) electron microscope using EBSD phase composition analysis.

Figure 2 shows the structural test results of the Nb sample after SPS sintering obtained with a CrossBeam-540 electron microscope using EBSD phase composition analysis.

The structure was studied using an electron microscope using the EBSD method. For characterization, a longitudinal microsection was made, passing through the axis of the sample, thus forming a section. Prior to analysis, the polished cross-sectional surface of the sample was examined for the presence of gas bubbles and other discontinuities, which are typical defects in powder metallurgy. The range of small and medium magnifications up to ×8000 was used. No sintering defects or foreign inclusions were found on the studied thin section. Then, several arbitrary sections of the sample section were mapped for testing the technique. The EBSD map was taken at ×100 magnification, since the grains turned out to be quite large. The average grain diameter according to the statistics after processing RAW data in the Tango HKL Channel5 module for 53 grains was 158 µm, the average grain area was 26,580 µm^2^. When recognizing, 6.4% of zero solutions were obtained. According to the pole figures, the microstructure of the sample does not have any pronounced direction of orientation. All grain boundaries are high-angle and the grains are oriented in random directions. All grains are equiaxed with a small (also randomly oriented) eccentricity within 1.3–1.7.

It can be concluded that the grains have no signs of deformation in any direction, which indicates that the process of powder preparation and sintering was carried out correctly. The resulting microstructure is indistinguishable from that of conventionally produced metal after recrystallization annealing [6,44,45].

### 3.3. Results of Mechanical and Rheological Tests on Nb Samples Obtained Using the SPS Method. Comparison of Results with the Traditional Manufacturing Method

**Figure 3:**
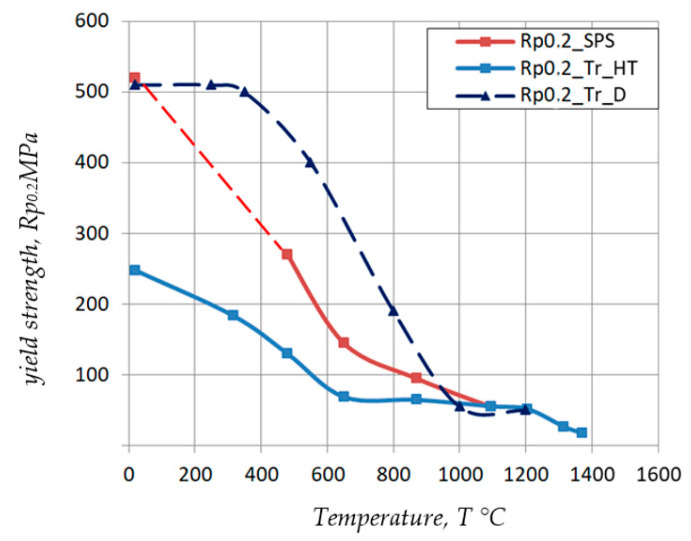
Results of tests of the conventional yield strength R_p0.2_ versus the temperature of specimens obtained by the new technology—sintered by the SPS method (Rp_0.2__SPS), obtained by the traditional technology in a state after cold deformation (R_p0.2__Tr_D) and after recrystallization annealing (R_p0.2__Tr_HT).

Figure 3 shows the results of tests on the conventional yield strength R_p0.2_ of samples obtained using the new technology—sintered using the SPS method (R_p0.2__SPS), those obtained using the traditional technology in the cold-formed state (R_p0.2__Tr_D) and after recrystallizing annealing (R_p0.2__Tr_HT). The tests were conducted over a wide temperature range from 20 °C to 1400 °C.

Based on the analysis of the mechanical properties results obtained from compression tests, at T = 20 °C and strain rate $\dot{\varepsilon }$ = 10^−3^ s^−1^, it was found that the conventional yield strength value R_p0.2_ for samples obtained according to the new technology (R_p0.2__SPS) corresponds to the yield strength R_p0.2_ of samples obtained according to the traditional technology in the cold deformed state (Rp_0.2_Tr_D_) and is almost twice as high as in the recrystallized state (Rp_0.2__Tr_HT).

As the temperature of the samples increases to T ≈ 300 °C, the value of the conventional yield stress (Rp_0.2__Tr_D) does not change and is 510 MPa. This temperature value is insufficient to activate thermally activated processes. In contrast, for samples (Rp_0.2__Tr_HT) and (Rp_0.2__SPS), the value of the conventional yield stress R_p0.2_ decreases, which is related to the absence of a deformed structure. As the temperature increases above T > 400 °C, softening processes begin to occur in the sample (R_p0.2__Tr_D) and the value of the conventional yield stress R_p0.2_ decreases. This decrease is observed in the temperature range up to 1000 °C.

Based on the analysis of the results of conducted tests, it can be concluded that the value of the conventional yield strength of the sample obtained using the new technology (Rp_0.2__SPS) is higher than the value of the conventional yield strength of the sample (after recrystallization annealing) produced using the traditional technology (Rp_0.2__HT). For specimens heated to temperatures T > 1000 °C, the values of the conventional yield stress for all specimens are the same.

**Figure 4:**
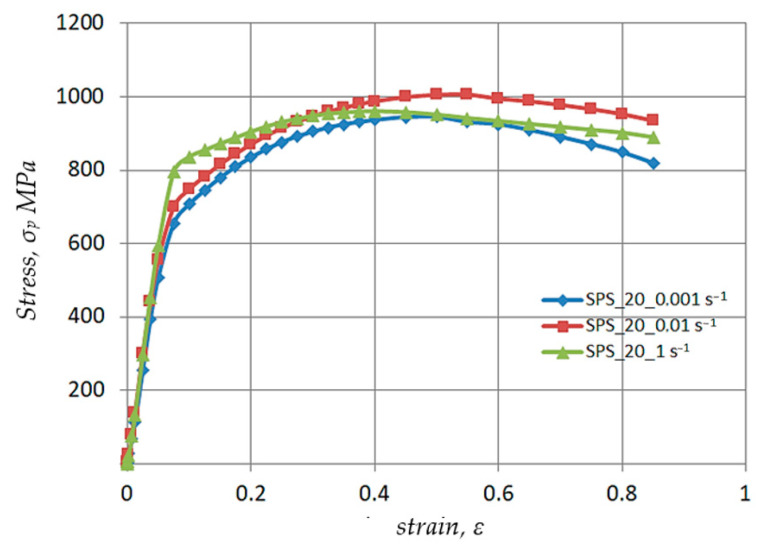
Test results of the effect of actual strain and strain rate έ = 0.001–1.0 s^−1^ at room temperature on the value of plasticizing stress σ_p_ of Ø10 × 12 mm Nb samples obtained according to the new technology—sintered by the SPS method.

**Figure 5:**
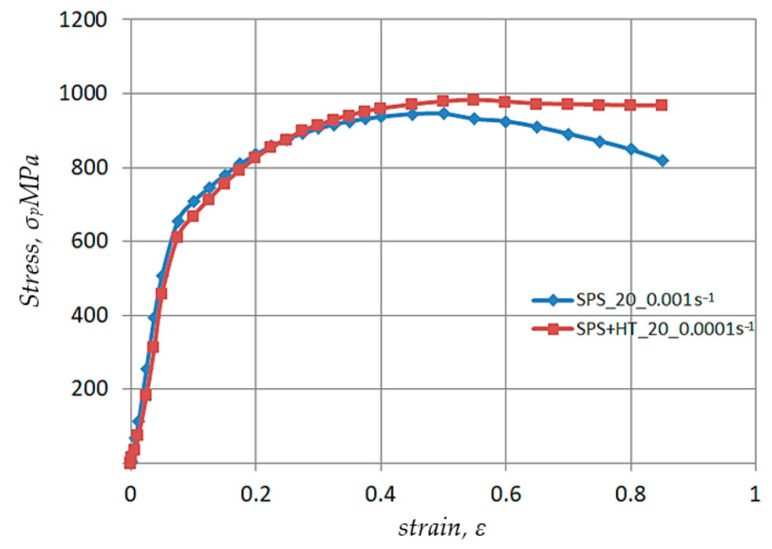
Results of investigations into the impact of the structure’s state (after SPS sintering as well as after recrystallization annealing) on the σ_p_-ε curves obtained during uniaxial compression of Ø10 × 12 mm Nb samples at strain rate έ = 0.001 s^−1^.

**Figure 6:**
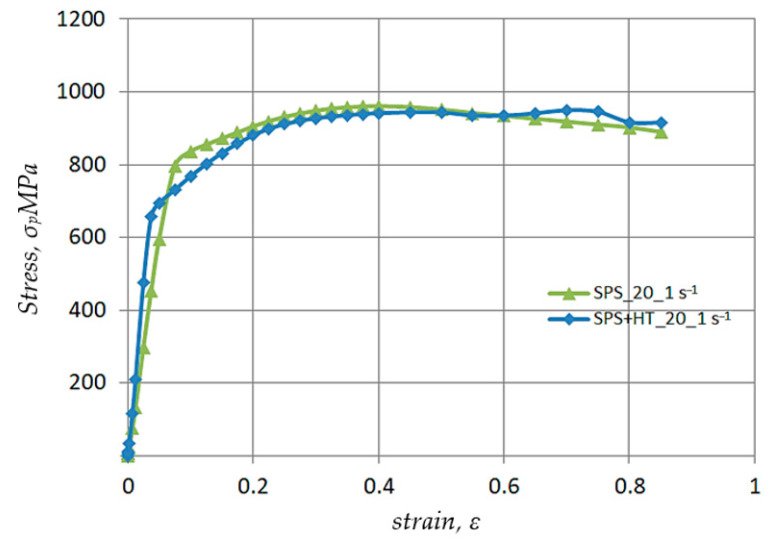
Results of investigations into the impact of the structure’s state (after SPS sintering as well as after recrystallization annealing) on the σ_p_-ε curves obtained during uniaxial compression of Ø10 × 12 mm Nb samples at strain rate έ = 1.0 s^−1^.

**Figure 7:**
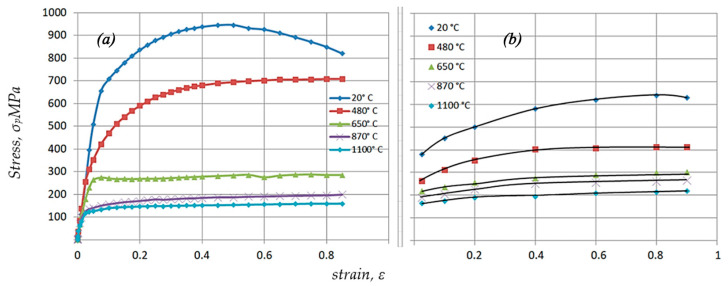
Comparative test results of the impact of temperature T and actual strain ε on the change in the plasticizing stress σ_p_ of Ø10 × 12 mm Nb specimens at strain rate έ = 0.001 s^−1^: (**a**) after sintering using the SPS method; (**b**) traditional technology.

Figure 4, Figure 5, Figure 6 and Figure 7 show the test results of the impact of thermomechanical parameters on the plasticizing stress o_p_. The results were obtained by performing swelling tests on Ø10 × 12 mm cylindrical samples using a Gleeble 3800 plastomer in a strain rate range έ = 0.001–1.0 s^−1^ and temperatures T = 20–1100 °C. The plastometric test parameters were chosen based on those used in actual technological processes such as forging, hot pressing and cold rolling [36,37,38].

The results of testing the impact of strain rate έ = 0.001–1.0 s^−1^ at room temperature are shown in Figure 4. The data analysis shows that as the strain rate increases from έ = 0.001 to έ = 0.01 s^−1^, the value of the strengthening factor and the value of the plasticizing stress o_p_ increases. Furthermore, the difference increases as the strain value ε rises. For a strain ε = 0.40, the difference is ~5.0%, and for ε = 0.80 the difference is ~11.0%. The maximum observed in the yield curves shifts with the increase in the strain rate έ to 0.01 s^−1^ from ε = 0.50 to ε = 0.55. This is due to the hindered plastic flow of the metal. Thus, if at έ = 0.001 s^−1^ all of the strain occurs by sliding, at higher strain rates twins appear in the structure and require more energy to activate their displacement [46].

As the strain rate increases to έ = 1.0 s^−1^, the character of the plasticizing yield curve o_p_-ε changes. For actual strain ε = 0.10, an increase in the plasticizing stress σ_p_ of approximately 11% is observed. Then, as the actual strain increases to ε = 0.3, the values determined by the plastic flow curve σ_p_-ε are the same as the values determined by the flow curve σ_p_-ε obtained for strain rate έ = 0.01 s^−1^. As the strain rate έ increases from 0.01 to 1.0 s^−1^, the maximum on the yield curve shifts from ε = 0.55 to ε = 0.40. For the actual strain ε = 0.60, the value of the plasticizing stress σ_p_ in the flow curve σ_p_-ε obtained at a strain rate έ = 1.0 s^−1^ decreases by ~6% compared to the curve obtained for a strain rate έ = 0.01 s^−1^. This course of the plastic flow curve results from the occurrence of a significant thermal effect of plastic deformation ∆T. During tests using the swelling method, the occurrence of a thermal plastic deformation effect was found on the surface of the cylindrical sample, the temperature rise being ∆T = 170 °C. As a result of the temperature increase, deformational softening processes were activated, causing a premature decrease in the plasticizing stress σ_p_. A decrease in σ_p_ at a strain ε greater than 0.6 in all presented cases is associated with a loss of stability in the macro-center of the plastic flow location.

Figure 5 and Figure 6 show the results of the strain rate and strain value effect on the plasticizing stress value of Ø10 × 12 mm Nb specimens obtained by sintering using the SPS method and after recrystallization annealing.

The data in Figure 5 shows that, due to recrystallization annealing, the value of the conventional yield stress R_p0.2_ decreases from 520 MPa to 490 MPa. Reducing the value of the conventional yield strength R_p0.2_ is advantageous in further stages of the forming process, e.g., by cold rolling or drawing, from the perspective of reducing energy costs and increasing tool life. It is also important to note that the recrystallization annealing resulted in an approximately 4.0% increase in the sample’s strength (UTS). Simultaneously, it is worth noting the section of determined plastic flow on the σ_p_-ε flow curve. Thanks to this property, under cold plastic processing conditions the recrystallized metal has a greater yield stress reserve.

The flow curves σ_p_-ε of the samples obtained through SPS sintering and after recrystallization annealing, obtained at a strain rate έ = 1.0 s^−1^, are generally similar, except in the range for which the actual strain ε < 0.2. The use of recrystallization annealing lowers the value of the strain-hardening coefficient, with plastic flow of the metal commencing at a stress of 670 MPa, compared to 750 MPa for samples obtained directly through SPS sintering (without recrystallization annealing).

Figure 7 shows the results of tests on the effect of temperature T and applied strain ε on the change in plasticizing stress σ_p_ of Ø10 × 12 mm Nb specimens obtained by SPS sintering (Figure 7a) and traditional technology (Figure 7b) for a strain rate έ = 0.001 s^−1^.

The data in this figure shows that when the temperature is increased from 20 °C to 1100 °C, the plasticizing stress σ_p_ decreases. Thus, when the temperature is increased to T = 480 °C and the strain ε = 0.4 is set, the plasticizing stress σ_p_ is reduced by ~27% (Figure 7a). Furthermore, note the change in the characteristics of the σ_p_-ε curve course. In the deformation area ε > 0.6, the course of the σ_p_ flow curve reaches a set state, which can be explained by the increasing effect of temperature on the activation of softening processes.

As the strain temperature increases, not only does the plasticizing strain decrease, but the nature of the σ_p_-ε curves also changes. Already in the region of small deformations ε < 0.1, the occurrence of a steady course of the σ_p_ curve can be ascertained, indicating an intensive course of dynamic metal softening processes [26]. When increasing the sample temperature to 650 °C for a given strain ε = 0.4, a decrease in the plasticizing stress σ_p_ of ~60% is observed, when increasing to 870 °C a decrease of 33% is observed, and at 1100 °C a decrease of 18% is observed.

The plasticizing stress values for the sample obtained by conventional technology differ from those of the samples obtained using the SPS method (Figure 7b). A particularly large difference is noticeable at T = 20 °C and T = 480 °C. Samples obtained using the traditional method have a significantly lower conventional yield strength R_p0.2_ and ultimate strength (UTS). Thus, at T = 20 °C and strain ε = 0.4, the difference in plasticizing stress σ_p_ is ~38%. As the temperature increases above T > 480 °C, there is a decrease in the difference between the σ_p_-ε flow curves achieved for samples obtained using the SPS method (Figure 7a) and samples obtained using conventional technology (Figure 7b).

**Figure 8:**
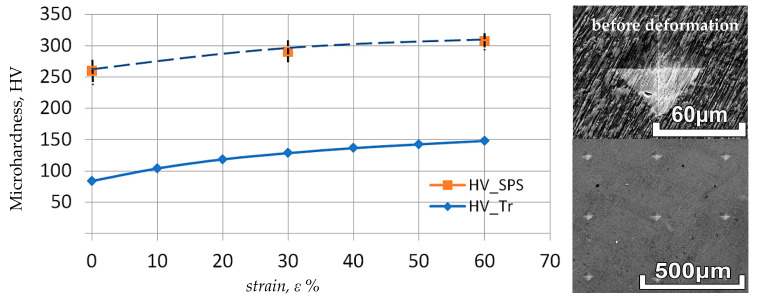
Results of microhardness tests of Nb samples obtained by the SPS method (HV_SPS) and by the traditional method (HV_Tr) depending on the applied strain value.

Figure 8 shows the results of microhardness tests on the samples as a function of the applied strain. The results shown in Figure 8 confirm the occurrence of significant differences in the properties of the metal obtained through the different methods. The microhardness value of samples obtained by the SPS method is more than double that of samples obtained by the traditional method.

The difference in the mechanical properties of the test samples obtained by SPS sintering and those obtained by traditional technology may be due to the higher density of the products obtained by the new technology. Furthermore, it is impossible to exclude the curing effect of the presence of gaseous impurities, which are less effectively removed compared to the traditional, more expensive vacuum arc melting of ingots.

Simultaneously, the metal obtained according to the new technology is characterized by greater strength and can be used for structural components, while retaining an additional safety margin compared to products obtained according to traditional technology.

Based on the metallographic results, it was noted that the microstructure obtained is comparable to that of a metal produced in the traditional method, after recrystallization annealing.

Additional recrystallization annealing reduces the yield strength R_p0.2_ and increases the yield stress, which is useful for further plastic processing.

## 4. Final Conclusions

1. The new SPS sintering technology for the manufacture of Nb blanks has a significantly shorter production cycle, compared to the traditional method. Additionally, the use of the SPS method reduces the cost of semi-finished product manufacture due to a reduction in process time and the elimination of waste associated with mechanical and chemical processing.
2. Based on an analysis of the test results of the samples (Ø20 × 30 mm, Ø30 × 11 mm, Ø40 × 12 mm) made using the SPS method, it was found that the material density obtained was close to the theoretical density. The microstructure obtained is comparable to that of samples obtained through the traditional method after recrystallization annealing. The average grain diameter was 158 µm and the average grain area was 26,580 µm^2^. The resulting grains are equiaxial with a slight eccentricity in the range of 1.3–1.7.
3. Comparative studies of Nb specimens obtained by SPS sintering and according to the traditional technology show that samples obtained by the new technology have higher mechanical properties R_p0.2_ and R_m_ and hardness, especially deformed at low temperatures. This difference ensures that an additional safety margin is obtained for construction materials with Nb.
4. After recrystallization annealing at T = 1100 °C for 30 min, a decrease in the yield strength R_p0.2_ occurs, which has a beneficial effect on subsequent plastic processing, including cold pilger rolling and drawing. In addition, this heat treatment causes an increase in the resistance of the metal to the location of plastic flow, which will increase the metal’s yield reserve.

## Figures and Tables

**Table 1:** Chemical composition of Nb powder, wt. %.

**Nb**	**Ta**	**Fe**	**Ti**	**Si**	**W**	**C**	**O**	**H**	**N**	**Ni**	**Mo**	**Al**	**Mg**
99.7	0.06	0.002	0.001	0.002	0.003	0.003	0.2	0.002	0.02	0.001	0.0025	0.001	0.001
**Mn**	**Co**	**Sn**	**Cu**	**Zr**	-	-	-	-	-	-	-	-	-
0.001	0.001	0.001	0.003	0.001	-	-	-	-	-	-	-	-	-

**Table 2:** Test parameters for Nb specimens tested using a Gleeble 3800 plastomer.

έ, s^−1^	Test Temperature, °C					
	20		480	650	870	1100
	After SPS	After SPS i obr. term.	After SPS	After SPS	After SPS	After SPS
10^−3^	+	+	+	+	+	+
10^−2^	+	−	−	−	−	−
1	+	+	−	−	−	−

**Table 3:** Values of density ρ and hardness HV of Nb powder samples obtained after sintering using the SPS method in the temperature range T = 1800–2200 °C.

Sintering Temperature, T °C	Density, g/cm^3^	Hardness, HV
2200	8.56	255 ± 15
2000	8.56	260 ± 15
1800	8.54	240 ± 18
Theoretical density Nbρ = 8.57 g/cm^3^		

## Data Availability

The original contributions presented in the study are included in the article, further inquiries can be directed to the corresponding author.
